# Pattern Classification Using an Olfactory Model with PCA Feature Selection in Electronic Noses: Study and Application

**DOI:** 10.3390/s120302818

**Published:** 2012-03-01

**Authors:** Jun Fu, Canqin Huang, Jianguo Xing, Junbao Zheng

**Affiliations:** 1 College of Computer Science & Information Engineering, Zhejiang Gongshang University, Hangzhou 310018, China; E-Mails: junfu@zjgsu.edu.cn (J.F.); huangcanqin@163.com (C.H.); 2 College of Informatics and Electronics, Zhejiang University of Science and Technology, Hangzhou 310018, China; E-Mail: zhengjunbao@zstu.edu.cn

**Keywords:** artificial neural network, olfactory model, feature selection, principal component analysis, pattern classification, electronic nose

## Abstract

Biologically-inspired models and algorithms are considered as promising sensor array signal processing methods for electronic noses. Feature selection is one of the most important issues for developing robust pattern recognition models in machine learning. This paper describes an investigation into the classification performance of a bionic olfactory model with the increase of the dimensions of input feature vector (outer factor) as well as its parallel channels (inner factor). The principal component analysis technique was applied for feature selection and dimension reduction. Two data sets of three classes of wine derived from different cultivars and five classes of green tea derived from five different provinces of China were used for experiments. In the former case the results showed that the average correct classification rate increased as more principal components were put in to feature vector. In the latter case the results showed that sufficient parallel channels should be reserved in the model to avoid pattern space crowding. We concluded that 6∼8 channels of the model with principal component feature vector values of at least 90% cumulative variance is adequate for a classification task of 3∼5 pattern classes considering the trade-off between time consumption and classification rate.

## 1. Introduction

Some bionic analytical instruments such as electronic nose (eNose) and electronic tongue (eTongue) generally consist of an array of cross-sensitive chemical sensors and an appropriate pattern recognition (PARC) method for automatically detecting and discriminating target analytes [1–3]. The PARC methods have become a critical component in the successful implementation of such human sense-mimicking systems [4]. Many PARC methods have been introduced into e-noses, falling into three general categories: multivariate statistical approaches [5], artificial neural network (ANN) approaches [6,7], and neuromorphic approaches [8,9]. Among those methods, biologically-inspired models and algorithms are just beginning to emerge, but are considered as promising sensor array signal processing methods for future eNose development.

Besides, feature selection or feature extraction is also an important issue for developing robust PARC models in machine learning. The outputs of sensor arrays are usually time series. Some features should be extracted to represent the original signals for PARC systems. In some cases, not all elements in feature vector obtained from the preprocessing stage are essential for classification due its high-dimensionality and redundancy [10]. For example, hundreds of variables per measurement (m/z fragments) are available as “pseudo sensor” in a mass-spectrometry-based electronic nose (MS-based eNose) [11,12]. And a significant number of them are noisy or highly correlated. Thus feature selection techniques, which finding a small set of parameters that somehow represent the overall information, have become an unavoidable preprocessing step for developing robust PARC models. The obvious advantages of those are reduction in classifier training and testing time, and overcoming the curse of dimensionality, as well as improving classifier accuracy sometimes. Many feature selection techniques are used for eNose applications, such as curve fitting [13], discrete wavelet transform [14], principal component analysis (PCA) [15,16], linear discriminant analysis[17] and so on.

In our previous work [18–20], we introduced an olfactory neural network called the KIII model for pattern recognition in electronic noses. The distributed open-ended structure of the KIII model is compatible of any dimension of input vector; however, more running time is costly because six more second-order ordinary differential equations will be added into the system when one channel of the model is extended, so for a given classification task it is always advisable to reduce the dimensions of input vectors as much as possible. This paper is concerned with the relationship between classification performance of the KIII model and the outer “data” factor, *i.e.*, the dimension of input feature vector, as well as the inner “structure” factor, *i.e.*, the amount of its corresponding parallel channels. The PCA technique is taken as example for dimension reduction and selection for two data sets (three classes of wine and five classes of green tea, respectively). The study may be helpful to determine the input feature vector in high-dimensional data (e.g., MS-based eNose data) and its parallel channels as applying the KIII model for pattern recognition.

## 2. Materials and Methods

### 2.1. KIII Olfactory Model

**Figure 1.**
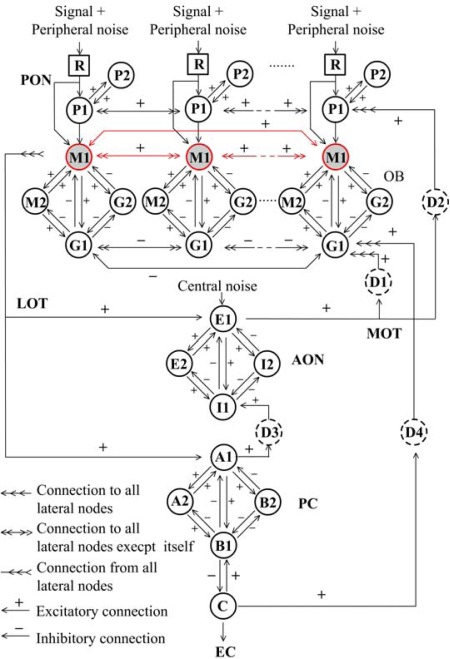
The topological structure of the KIII olfactory model [19]. Notes: The lateral connection weights of M1 nodes (red arrows) are adjustable for learning, while the others (black arrows) are usually fixed for system stability.

Over the last decades of study on mammalian olfactory system, Freeman and his colleagues [21–23] developed an olfactory model entitled KIII, which appropriately describes the whole olfactory pathway from sensory neuron to olfactory cortex, as well as offering a probable interpretation of principle of biological olfaction in the concept of mesoscopic neurodynamics [24]. The KIII model is a massively parallel architecture with multiple layers coupled with both feed-forward and feedback loops through distributed delay lines, as shown in Figure 1. External stimulus signals from receptors (R) transmit to periglomerular cells (P) and then to the olfactory bulb (OB) layer via the primary olfactory nerve (PON) in parallel. The OB layer consists of a set of mutually coupled neural oscillators, each being formed by two granule cells (G) and two mitral cells (M). Then the total output of all M1 nodes transmits via a lateral olfactory tract (LOT) to the anterior olfactory nucleus (AON) and prepyriform cortex (PC) layer, which provides the final output of the system to other parts of the brain from deep pyramidal cells (C), meanwhile back to the OB and AON layers [8].

The dynamics of every node can be described by a 2nd-order ordinary differential equation (ODE) as follows:

(1) $$\frac{1}{a\cdot b}\left[x_{i}^{\prime \prime }(t)+(a+b)x_{i}^{\prime }(t)+a\cdot b\cdot x_{i}(t)\right]=\sum_{j\neq i}^{N}\left[W_{\mathrm{ij}}\cdot Q(x_{j}(t),q_{j})\right]+I_{i}(t)$$

where *x_i_*(*t*) and *x_j_*(*t*) respectively represent the state variable of the *i*-th and the *j*-th node connected with each other, and W*_ij_* indicates the connection weight from *j* to *i*. I*_i_*(*t*) is an external input signal to the *i*-th node. The parameters *a* and *b* reflect two rate constants. *Q*(·) is a sigmoid function derived from the Hodgkin-Huxley equation and evaluated by experiments [25]:

(2) $$\begin{array}{l} Q(x_{j}(t),q)=\{\begin{array}{ccc} q\cdot (1-\text{exp}(-(\text{exp}(x(t)/q\;)\;)-1) & , & x(t)>x_{0}\\ -1 & , & x(t)<x_{0} \end{array}\\ x_{0}=\text{ln}(1-q\cdot \text{ln}(1+1/q)) \end{array}$$

The dynamics of the whole KIII system can be mathematically expressed by a set of ODEs. The parameters of W*_ij_* in the KIII model were determined by a set of reliable parameter optimization algorithms to ensure the model output electroencephalogram-like waveform as observed in olfactory systems [26]. For stability and robustness of the system, a low-level Gaussian noise was imported into R and AON to simulate peripheral and central sources of noise in olfactory systems, respectively [27]. Theoretical studies showed many dynamics behaviors in the model such as limit cycle, fixed point, quasi-periodic oscillation and chaos [22–27]. The optimized KIII model is not only considered as a mathematical model for neurobiology study, but also of broad interested for its good pattern learning and recognition potential.

### 2.2. Learning and Classification for KIII Model

**Figure 2.**
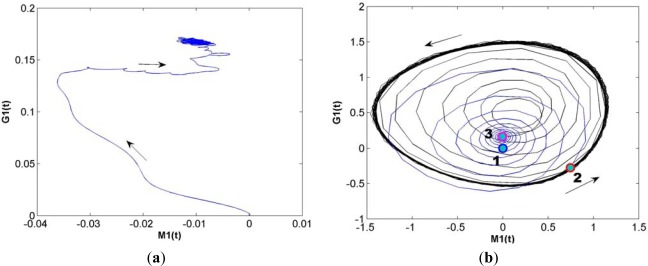
The trajectory in phase-space. (**a**) Before; (**b**) During stimulation.

The operation of learning and memorizing in the KIII model can be described as follows. The system with no stimuli is in a high dimensional state of spatially coherent basal activity, presenting a chaotic global attractor in phase space; while with external stimulus the system will soon turn to a γ-range of quasi-periodic burst, presenting a local basin of an attractor wing in phase space. Using some learning rules to adjust lateral connection weights in the OB layer, the model is able to remember a number of odor patterns. When a familiar odor presents again, its spatiotemporal pattern will soon transmit to a corresponding local attractor. Figure 2(a) shows the trajectory with M1 node and G1 node of the same channel in phase space without any stimuli (peripheral noise and central noise still exist). Figure 2(b) shows the trajectory before stimulation (blue curve of Note 1), during stimulation (black curve of Note 2) and after stimulation (black curve of Note 3). It can be seen that the system state changes quickly as stimuli change.

**Figure 3.**
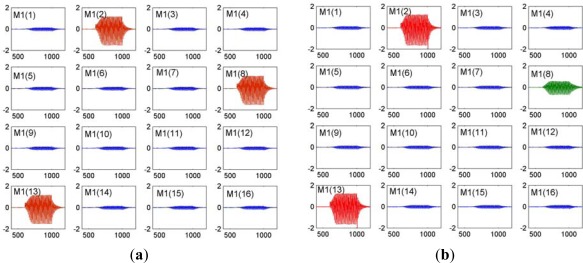
Binary experiments for (**a**) Pattern reproduce; (**b**) Pattern retrieval in a 16-channle KIII model with simple Hebbian learning rule.

Figure 3 shows pattern reproduce and retrieval of a 16-channle KIII model with simple Hebbian learning rule. Every subplot presents an amplitude modulated pattern (AM) output of each M1 node in the OB layer. Figure 3(a) is pattern output of learned pattern P = [0 1 0 0 0 0 0 1 0 0 0 0 1 0 0 0]. The consistent channel position and amplitude with pattern P demonstrate a memory recall. Figure 3(b) is pattern output of an incomplete version P’ = [0 1 0 0 0 0 0 0 0 0 0 0 1 0 0 0]. Compared with pattern P, there is an AM burst in input-absence channel M1(8) (green curve), demonstrating a good pattern recovery property.

Some learning rules were proposed for the KIII model, such as Hebbian reinforcement learning rule, global habituation rule, anti-Hebbian learning rule and local habituation rule [8,23,28,29]. In this paper a training algorithm combined both Hebbian reinforcement learning rule and global habituation rule is applied to adjust the lateral connection weights of M1 nodes in the OB layer, as (3):

(3) $$\begin{array}{c} \mathrm{If}\;\;\;\;M_{1}(i)>M_{m}\;\;\;\mathrm{and}\;\;M_{1}(j)>M_{m}\;\;\;\mathrm{then}\\ \mathrm{If}\;\;\;\;W_{\mathrm{ij}}<h_{\text{Heb}}\;\;\;\;\;\mathrm{then}\\ W_{\mathrm{ij}}^{\prime }=R_{\text{Heb}}*W_{\mathrm{ij}}\\ \mathrm{else}\;\;\;W_{\mathrm{ij}}^{\prime }=h_{\text{Heb}}\\ \mathrm{else}\;\;\;W_{\mathrm{ij}}^{\prime }=h_{\text{hab}}*W_{\mathrm{ij}} \end{array}$$

When the activities of the *i*-th and the *j*-th M1 node are larger than the mean activity of all M1 node M*_m_* in the OB layer, their connection weights are strengthened by *R_Heb_* (R*_Heb_* > 1); otherwise their connection weights decrease at the habituation rate h*_hab_* (h*_hab_* < 1) and eventually diminish asymptotically toward zero after several training cycles. h*_Heb_* is for value restriction of connection weights. The activity is measured by the standard deviation of AM burst in M1 node. From the view of artificial neural network, it is an unsupervised learning method and the goal is to make the connection weight space converge for small enough.

At the end of learning, the connection weights are fixed and the cluster centroids of every pattern are determined. While inputting a new sample from testing set, the Euclidean distances from the corresponding activity vector to those training pattern cluster centroids are calculated, and the minimum distance determines the classification.

All model implementations in this paper were carried out in MATLAB 7.5 (The MathWorks^®^ Inc., Natick, MA, USA) on a Lenovo computer (Pentium^®^ Dual CPU 1.86 GHz and RAM 2 GB) running Windows XP (Microsoft^®^ Corp., Redmond, WA, USA).

### 2.3. E-nose Instrumentation

**Figure 4.**
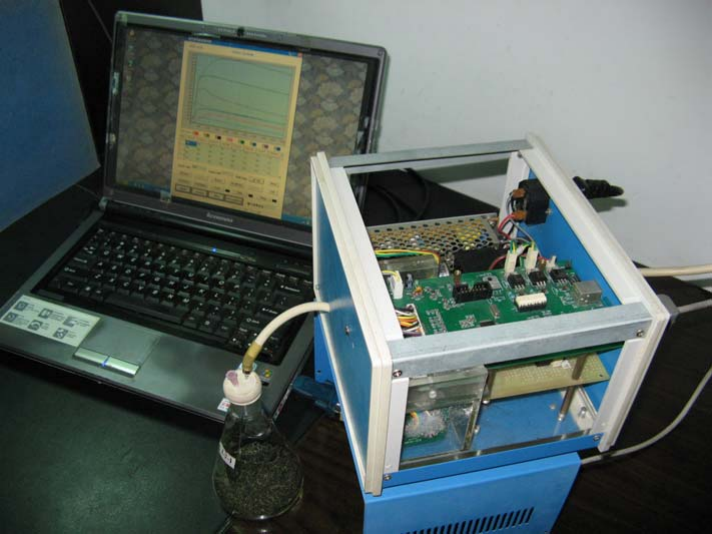
Photo of the experimental set-up with the customized electronic nose system.

A homemade electronic nose system has been developed for data acquisition, as illustrated in Figure 4. Eight MOS Taguchi-type gas sensors (TGS832, TGS880, TGS800, TGS822, TGS826, TGS825, TGS816 and TGS812) were purchased from Figaro Engineering Inc. (Osaka, Japan). They are arranged in two PCB boards with corresponding voltage divider circuits, and then fixed in a sealed gas chamber with a volume of 315 mL. Two micro-bumps (0.5 L/min) are connected to the chamber for aroma sampling and exhausting. The measurement are controlled, monitored and recorded by homemade Delphi software in a computer which connected to a microprocessor-based circuit (MSP430 with a 12-bit ADC) via a RS-232 cable.

## 3. Results and Discussion

### 3.1. Case I: Wine Classification

To demonstrate the classification performance of the KIII model with respect to dimension varying of input feature vector, a wine data set provided by UCI Machine Learning Repository [30] was used in this paper. These data are the results of a chemical analysis of wines grown in the same region in Italy but derived from three different cultivars. The analysis determined the quantities of 13 constituents found in each of the three types of wines. All data are 13-dimension with three classes defined by the three cultivars. The data sets are very large, with 59, 71 and 48 samples per class labeled as Class 1, Class 2 and Class 3. The detail on the data set can be found in [30].

Selecting an appropriate set of features which is optimal for a given classification task is one of the most important issues in machine learning. Many techniques such as principal component analysis and independent component analysis (ICA) produce a mapping between the original feature space to a lower dimensional feature space, and are usually proposed for dimension reduction and feature selection.

**Figure 5.**
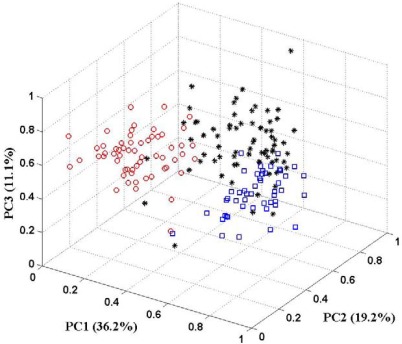
The 3D-PCA plot of three classes of wine derived from three different cultivars. Note: Symbols: ^O^-Class 1; *-Class 2 and □-Class 3.

In this paper, the PCA technique is used for feature selection. The aim is to pick out patterns in multivariate data and reduce the dimensionality of the input vector without a significant loss of information. PCA can also help to get an overall view of these data through giving an appropriate visual representation with fewer dimensions. Figure 5 shows the 3D-PCA plot of three classes of wine. Examining the PCA plot, the first three principal components (PCs) account for 66.5% of the variance of the data. There is not a good clustering of three classes in 3D plot. Some samples of one class drift into feature spaces of other classes. It implies that the first three principal components do not represent sufficient information for the data set. The some other of principal components should be put in to preserve more of the relevant information of the original data. There are some guidelines for determining the optimal amount of PCs [31,32]. In the following part of this section, more than three principal components will present to the KIII model as feature vector. And the correct classification rates are calculated to make sure how many principal components are sufficient.

**Figure 6.**
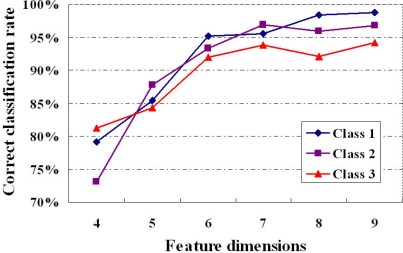
The correct classification rate of three classes with respect to PC numbers.

**Figure 7.**
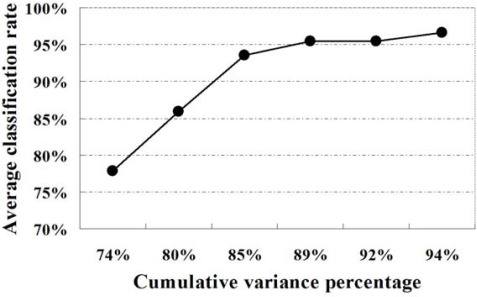
The average correct classification rate with respect to cumulative variance percentage of principal components.

Five samples in each class of wine data set were randomly chosen for training set and the others were used for testing. The first *n* principal components are selected as input feature vector for *n*-channel KIII model. The parameters of the KIII model in this paper are h*_hab_* = 0.5487, h*_Heb_* = 0.0395, R*_Heb_* = 1.5 and the others are from [27]. The 4th-order Runge-Kutta method with a fixed step of one (1,200 steps total) was applied for numerical integration of the ODEs. The average correct classification rates of ten trials are presented in Table 1, as well as in Figure 6. From the plot, it is very clear that the classification performance of the KIII model gradually become better and better as PC numbers increasing. Figure 7 shows the average correct classification rates of three classes with the increasing of cumulative variance percentage (CVP). Roughly speaking, more than 85% of CVP would represent sufficient information of the data set, and could get 90% of correct classification rate. However, the running time of KIII model implementation increased correspondingly. Considering the trade-off between time consumption and classification rate, the optimal dimension of feature vector is about seven in this classification task.

### 3.2. Case II: Tea Classification

**Figure 8.**
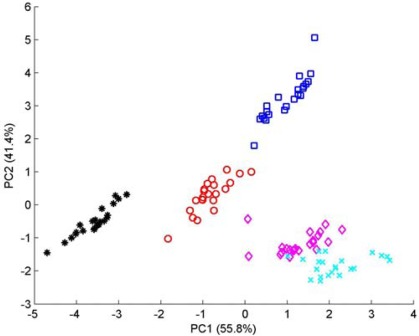
The 2D-PCA plot of five classes of green tea derived from five different provinces of China. Note: Symbols: ^O^-class 1; *-class 2; □-class 3; ♦-class 4; x-class 5.

Five brands of commercial green tea derived from five different provinces of China, *i.e.*, Anhui, Henan, Hubei, Sichuan and Zhejiang (labeled as Class 1 to Class 5 in turn), represent different cultivars and tea-making process technology. For each brands of green tea, 22 measurements were made through the customized e-nose instrumentation described in Section 2.3. Every measurement lasted more than 400 sec with a sampling rate of 20 Sa/sec, and yielded a large data matrix. PCA was also used here to investigate how the response vectors from sensor arrays cluster and reduce the dimensionality of the raw data. Figure 8 shows the 2D-PCA plot of five classes of green tea. Examining the PCA plot, the first two principal components account for 97.26% of the variance of the data-set (PC1 and PC2 accounted for 55.83% and 41.43% of the variance, whereas PC3 and PC4 accounted for 1.96% and 0.53%, respectively). Despite the slight overlap of two clusters, we can easily observe an excellent discrimination of the five kinds of green tea.

According to the experience of Section 3.1, in this case, only the first two principal components are sufficient as selected features to train KIII model. The verification experiment was carried out as follows. Five samples in each class of tea data set were randomly chosen for training set and the others were used for testing. The first two principal components were selected as input feature vector for a 2-channel KIII model. The parameters of the model and the implementing method were the same as described above. The average correct classification rates of ten trials are presented in Table 2. Exceeding our expectations, the average classification rates of five classes are only 9.88%. Some classes are completely confused with others. When more than two principal components presented to the KIII model, the average classification rates rose strikingly from 18.35% to 87.88%, and then slowly and steadily at about 95.06%. It suggests that CVP is not a unique factor to effect KIII model’s classification performance.

As described in Section 2.2, the KIII model acts as an associative memory capable of storing previously trained patterns by means of Hebbian rule at OB layer, and recovering incomplete or corrupted pattern. From a neurodynamics viewpoint, there is a global chaotic attractor composed of a central part and multiple wings in the KIII system, and the functions above work through the transition back and forth between the central part and one wing or between the wings. When parallel channels of the KIII model reduce, there will not enough state space to represent that kind of attractor, *i.e.*, different pattern classes. That is to say, pattern volume of the KIII model is related to the amount of its parallel channels. It is concluded that six channels is adequate for the classification task of five pattern classes in this case.

## 4. Conclusions

In this paper, the classification performance of a bionic olfactory model called KIII with respect to outer factor (the dimension of input feature vector) and inner factor (the amount of its parallel channels) was investigated, through recognition of three classes of wine derived from three different cultivars and five classes of green tea derived from five different provinces of China. In the first case, the PCA plot implied that the first three principal components do not represent sufficient information for the database, so more PCs were put in to feature vector and the corresponding classification rates were calculated. The results showed that classification performance of the KIII model gradually becomes better and better as PC numbers increase. Considering the trade-off between time consumption and classification rate, a PC feature of seven (about 90% of cumulative variance percentage) is an appropriate dimension for this special task. In the second case, the first two PCs (about 97% of cumulative variance percentage) were selected as input feature vector for a 2-channel KIII model. The poor results suggested that cumulative variance percentage is not the only factor to affect the KIII model’s classification performance. The results showed that classification performance of the KIII model gradually become better and better as its parallel channels extend, so we concluded that 6∼8 channels of the model with principal component feature vector values of at least 90% cumulative variance is adequate for a classification task of 3∼5 pattern classes considering the trade-off between time and classification rate. The study may be helpful to determine the input feature dimension and its parallel channels as applying the KIII model for pattern recognition (not limited to eNose applications). Future works will be addressed on analyzing higher-dimensional MS-based electronic nose data using the model.

## Figures and Tables

**Table 1.** The classification performance of the KIII model with the increase of the dimension of input feature vector.

**PCs**	**4**	**5**	**6**	**7**	**8**	**9**
Class 1	79.19	85.45	95.14	95.56	98.34	98.82
Class 2	73.08	87.82	93.29	96.96	95.91	96.73
Class 3	81.25	84.35	92.03	93.83	92.07	94.14
Average	77.84	85.87	93.49	95.45	95.44	96.57
CVP	73.60	80.16	85.10	89.34	92.02	94.24

Note: all classification rate and PCA values in the table are expressed as percentages.

**Table 2.** The classification performance of the KIII model for five kinds of green tea with the increase of its parallel channels.

**PCs**	**2**	**3**	**4**	**5**	**6**	**7**
Class 1	10.59	14.12	30.59	37.06	87.06	95.88
Class 2	10.00	18.24	25.88	35.29	86.47	94.12
Class 3	7.06	15.29	27.65	44.12	92.94	93.53
Class 4	12.35	21.76	27.06	41.76	87.06	94.12
Class 5	9.41	22.35	34.71	48.24	85.88	97.65

Average	9.88	18.35	29.18	41.29	87.88	95.06

CVP	97.26	99.22	99.75	99.91	99.96	99.99

Note: all classification rate and PCA values in the table are expressed as percentages.
